# Lattice Dynamics Study of Phonon Instability and Thermal Properties of Type-I Clathrate K_8_Si_46_ under High Pressure

**DOI:** 10.3390/ma9080616

**Published:** 2016-07-25

**Authors:** Wei Zhang, Zhao Yi Zeng, Ni Na Ge, Zhi Guo Li

**Affiliations:** 1 School of Science, Southwest University of Science and Technology, Mianyang 610064, Sichuan, China; zwphys@gmail.com; 2 College of Physics and Electronic Engineering, Chongqing Normal University, Chongqing 400047, China; 3 State Key Laboratory Cultivation Base for Nonmetal Composites and Functional Materials, Southwest University of Science and Technology, Mianyang 610064, Sichuan, China; genina911@163.com; 4 Laboratory for Shock Wave and Detonation Physics Research, Institute of Fluid Physics, Chinese Academy of Engineering Physics, Mianyang 621900, Sichuan, China; zhiguo_li@foxmail.com

**Keywords:** phonon spectrum, clathrate compounds, lattice dynamics, high pressure

## Abstract

For a further understanding of the phase transitions mechanism in type-I silicon clathrates K_8_Si_46_, ab initio self-consistent electronic calculations combined with linear-response method have been performed to investigate the vibrational properties of alkali metal K atoms encapsulated type-I silicon-clathrate under pressure within the framework of density functional perturbation theory. Our lattice dynamics simulation results showed that the pressure induced phase transition of K_8_Si_46_ was believed to be driven by the phonon instability of the calthrate lattice. Analysis of the evolution of the partial phonon density of state with pressure, a legible dynamic picture for both guest K atoms and host lattice, was given. In addition, based on phonon calculations and combined with quasi-harmonic approximation, the specific heat of K_8_Si_46_ was derived, which agreed very well with experimental results. Also, other important thermal properties including the thermal expansion coefficients and Grüneisen parameters of K_8_Si_46_ under different temperature and pressure were also predicted.

## 1. Introduction

**Figure 1:**
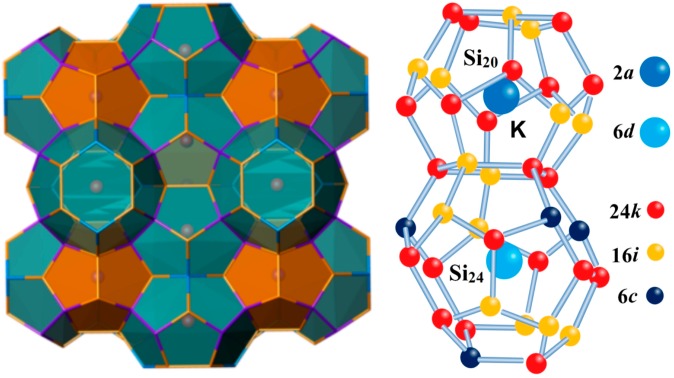
The sketch map of ball and stick representation of the type I clathrate K_8_Si_46_ with an illustration of Wyckoff sites of the structure.

Nanostructured type I clathrates are composed of two 20-atoms (small) and six 24-atoms (large) cages of group-IV elements which can host different kinds of atoms. The framework atoms admit partial substitution by other atomic species. Such good tailorability enables these clathrate compounds to have extensive potential applications in areas of superconductivity [1], wide-band-gap semiconductors [2], optoelectronics [3], magnetism [4], thermoelectric [5] and photovoltaics [6], etc. As illustrated in Figure 1, there are three distinct Wyckoff symmetry sites, i.e., 6*c*, 16*i* and 24*k* for the framework atoms. Encaptured metal atoms located at the center of small and large cages correspond to 2*a* sites and 6*d* sites, respectively. Since type-I Ba_8_Si_46_ had been realized in a multi-anvil press by the group of Yamanaka in 2000 [7], high pressure technique has opened up a new door to synthesize these kind of clathrates compounds and provided new ideas to explore the interaction mechanism between guest atoms and the host lattice. During the high pressure experiments in studying the stability and compressibility of these silicon clathrates, some clathrates doped with large guest atoms such as K_8_Si_46_ [8,9], Ba_8_Si_46_ [10,11,12,13,14,15], I_8_Si_44_I_2_ [16,17], Rb_6_Si_46_ [18] exhibit an abrupt pressure induced volume collapse transition while the overall crystal symmetry is preserved. Various mechanisms (e.g., the change of hybridization between guest atoms and frame cages [12] and an electronic topological transition [14,15]) for this high pressure phase transition have been proposed to explain this phenomenon for Ba_8_Si_46_. In our recent work [19], detailed investigations have been performed to study the effect of forming lattice vacancies on the mechanical and electronic properties of Ba_8_Si_46_ under high pressure. The results indicate that the compressbility of Ba_8_Si_46_ is governed by the Si framework and the pressure induced escape of host Si atoms would cause the sudden volume collapse during the compress, which is consistent with the explanation from Iitaka et al. according to their atomistic model [20]. A more coherent picture of the phase transition is given by the newest experimental study of the clathrate collapse in mixed Ba_8_(Si,Ge)_46_ clathrates which suggests that the volume collapse is a second order transition through a Landau modeling ,with a symmetry-breaking mechanism related to the Ba displacement initiated either by vacancy creation or by local distortion of the framework structure [21].

In the case of K_8_Si_46_, a noticeable change in the compressibility had been found at around 15 GPa followed by an abrupt change of volume at around 20 GPa. Above 32 GPa, the sample transformed into an amorphous phase. Earlier ab initio phonon band structure calculations using finite displacement method [22] had found that vibrational frequencies of the K atoms in the large cavities became imaginary at a pressure of 16 GPa which suggested a phase transition triggered by positional disordered guest atoms [8]. However, it is known that the supercell should be constructed in a phonon calculation within this method [22], which is obviously unbearable for an originally large primitive cell of K_8_Si_46_. Therefore, in their calculations, just primitive cell was supposed to be used. Although the primitive K_8_Si_46_ cell under ground state has a large lattice constant of somewhat more than 10 Å, the magnitude of the force constants beyond that distance is generally expected to be negligible. In a covalent semiconductor and complex structured system, the required cutoff radius should be larger than 10 Å as a rule, thus such a treatment may cause unreliable results when the K_8_Si_46_ cell is compressed under high pressure (the lattice constant of K_8_Si_46_ under 15 GPa is about 9.68 Å). For a confirmation of this point, we have also performed the phonon calculation within finite displacement method using a unit cell of K_8_Si_46_ and indeed found that a large number of imaginary frequencies appeared when the pressure was applied up to 15 GPa. So, in the present work, we employ the density functional perturbation theory (DFPT) [23,24] which avoids the use of a supercell and allows calculation of the dynamical matrix exactly at an arbitrary ***q*** vector to study the stability of K_8_Si_46_ under pressure. For a perfect crystal, the dynamical instability of the phonon is found to be originated from the framework of K_8_Si_46_ which is quite different to the results reported by J. S. Tse et al. [8]. From the obtained partial phonon density of the state, a clearer dynamic picture of guest K atoms and host lattice under high pressure is given. Besides, based on the phonon calculation, we derived the thermal properties of K_8_Si_46_ from quasi-harmonic approximation which were believed to offer a useful reference to design and synthetize a new ternary type-I Si clathrate based on K_8_Si_46_ with enhanced thermoelectric performance.

## 2. Computational Methods

Phonon dispersion spectrum was calculated by using the linear-response method [25,26] within the density functional perturbation theory (DFPT) [23,24]. The full phonon dispersion curves were obtained through Fourier interpolation. Norm-conserving pseudopotentials generated using the kinetic energy optimization scheme developed by Lin et al. [27] and Lee [28] were employed to describe the ion-electron interactions with an energy cutoff of 800 eV to expand the valence electronic wave functions. Monkhorst-Pack *k*-points mesh of 6 × 6 × 6 had been chosen. The electronic exchange-correlation interactions were treated within the local density approximation (LDA) [29]. During the pseudopotentials calculations, pseudo atomic calculations were performed for K (3*s*, 3*p*, 4*s*) and Si (3*s*, 3*p*). By employing the Parrinello–Rahman method [30,31], hydrostatic pressure load on crystal was realized within the variable cell approach. Both the cell parameters and the atomic internal coordinates were fully relaxed at each target external pressure by applying Broyden, Fletcher, Goldfarb and Shanno (BFGS) scheme [32]. All these total energy electronic structure calculations were carried out by Cambridge Serial Total Energy Package (CASTEP) code [33,34]. 

## 3. Results and Discussion

### 3.1. Phonon Spectra

Within the framework of DFPT method, phonon frequencies are computed as second-order derivatives of the total energy with respect to a given perturbation in the form of atomic displacements. The force constants matrix can be obtained by differentiating the Hellmann-Feynman forces on atoms with respect to ionic coordinates. Based on the interatomic force constants, we can obtain the phonon spectra by using Fourier interpolation with specific treatment of the long-range dipole-dipole interaction [35]. The phonon band structure calculations were performed up to 40 GPa with an interval of 10 GPa. A more careful calculation at 39 GPa was performed for determining the exact pressure value when the phonon became instable.

**Figure 2:**
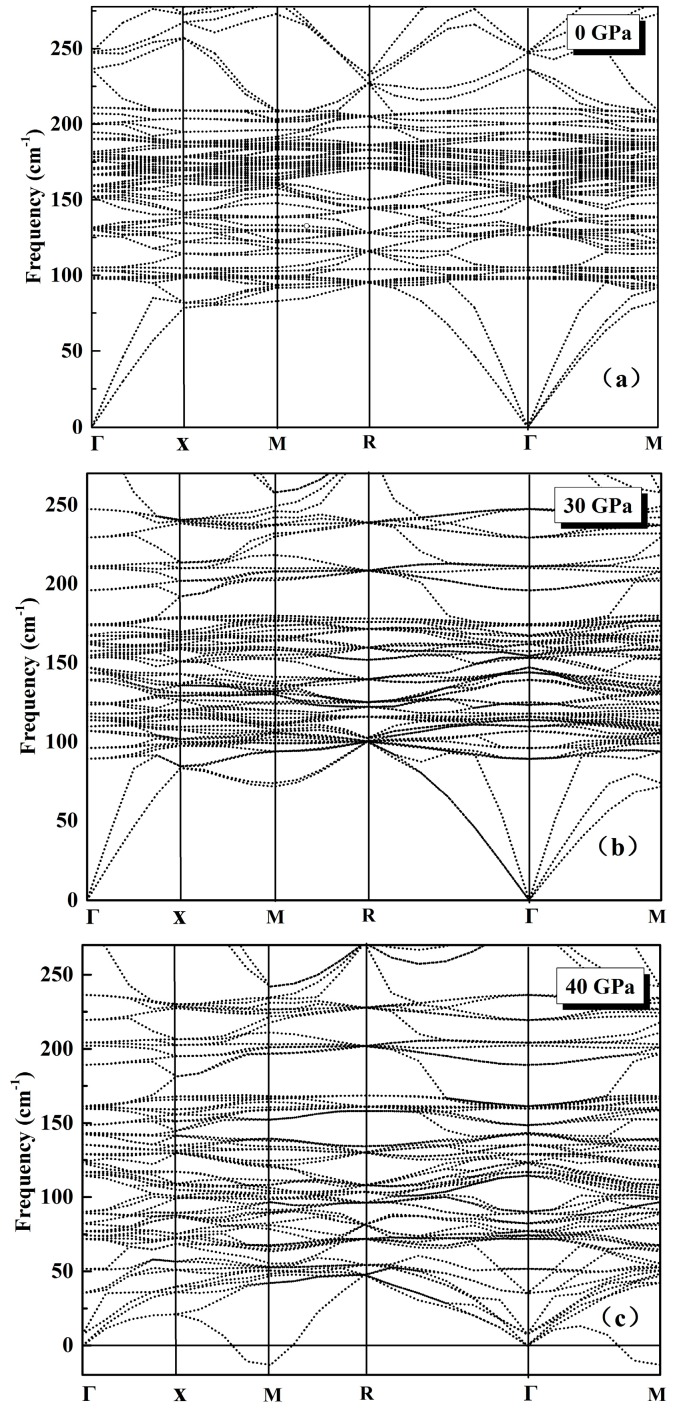
The phonon dispersion relation of K_8_Si_46_ at (**a**) 0 GPa; (**b**) 30 GPa and (**c**) 40 GPa.

**Figure 3:**
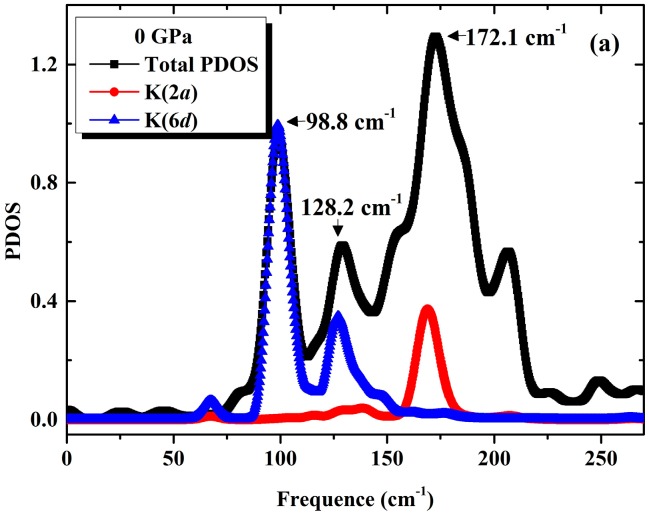

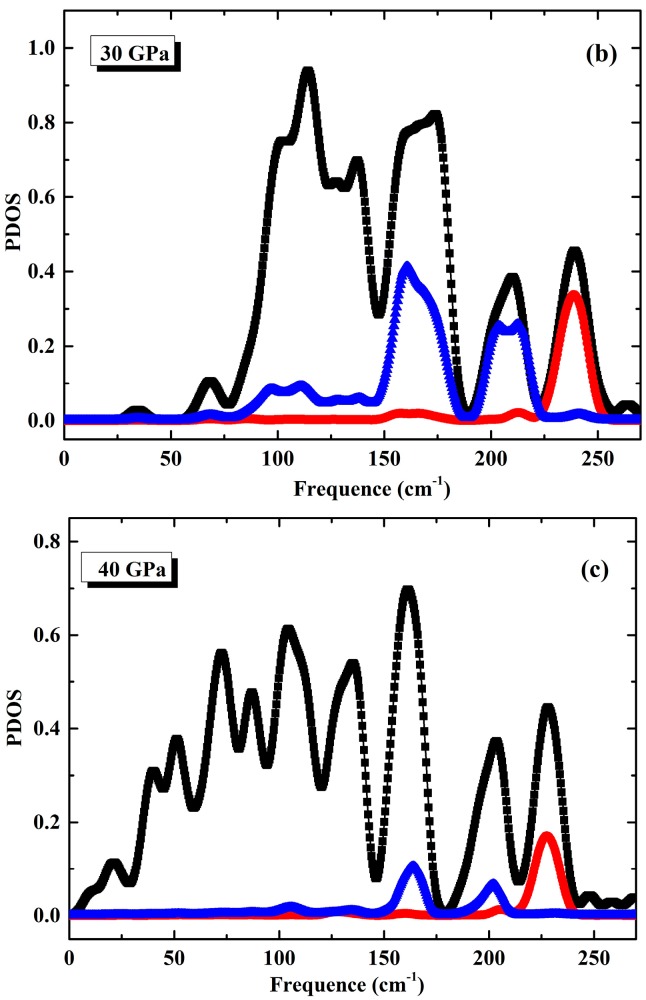
The partial phonon density of state of K_8_Si_46_ at (**a**) 0 GPa; (**b**) 30 GPa and (**c**) 40 GPa.

The obtained ground state phonon dispersion relations in K_8_Si_46_ crystal are shown in Figure 2a. Clear flat vibrational bands can be easily recognized in the low frequency region around 100 cm^−1^. By analysis of partial phonon density of state (PPDOS) as shown in Figure 3a, we find that these low frequencies are corresponding to a sharp peak located at about 98.8 cm^−1^ which are originated from the localized vibration of 6*d* sites K atoms at Si_24_ cages. Another somewhat weaker but still clear peak at 128.2 cm^−1^ also from K(6*d*) can be attributed to the asymmetry of the large Si_24_ cage which takes ellipsoidal shape. A similar phenomenon had also been observed in another type-I silicon-clathrate Na_8_Si_46_ reported by Li et al. [36]. Moreover, a strong and broad peak centered at 172.1 cm^−1^ is found to be contributed by the mixing vibrations of K atoms at Si_20_ cages and the framework Si atoms indicates the intense interaction hybridization between them, this is because the size of Si_20_ cage is much smaller than Si_24_ cage which yields a shorter interatomic distance of K-Si. Experimentally, the measured Raman spectrum of K_8_Si_46_ showed noticeable peaks at 94, 119 and 177 cm^−1^ related to the vibration of K atoms [9], which is consistent with our results. By inelastic neutron scattering, Mélinon et al. [37] and Reny et al. [38] obtained very similar vibrational spectrum of K_8_Si_46_ and found two identified peaks centered at about (100 cm^−1^, 170 cm^−1^) and (98 ± 2 cm^−1^, 161 ± 5 cm^−1^) respectively. Our calculated results perfectly reproduced the main characteristics of experimental observation. Under a pressure of 30 GPa, due to the shrinkage of the silicon cages under compress, the PPDOS shows strong mixing of K and Si vibrations. The frequency of K atoms’ motion at both 2*a* and 6*d* sites became higher, which indicates a further localization of these atoms, as shown in Figure 3b. However, it is noted that the appearance of massive low frequencies that originate from the framework atoms suggests a collective “soften” of Si-Si bonds which will finally make the host lattice unstable. As the pressure is applied at a value of 40 GPa, it can be seen clearly from Figure 2c that the frequencies around the M symmetry point become imaginary, which clearly shows the instability of clathrate framework. As illustrated in in Figure 3c, the PPDOS under 40 GPa also shows a dramatic reduction of frequencies from host lattice as presented in the phonon spectrum. Our calculation results show that a mechanical instability of the silicon framework is believed to be responsible for the pressure-induced volume collapse at about 20 GPa and subsequent amorphization at 32 GPa of K_8_Si_46_ observed experimentally.

However, it is noted that the unstable pressure given in the present work is obviously larger than the experimental observation. This is because our calculations are performed using a perfect K_8_Si_46_ crystal from the view of lattice dynamics without consideration of other possible transition mechanisms (e.g., vacancies formation [20], local symmetry-breaking [21] or an electronic topological transition [14,15], etc.) associated with the pressure collapse of the clathrate structure. If multi-mechanisms are involved in phase transition, the value of transition pressure would be affected a lot. For instance, the lattice vacancies are actually very likely to be produced in these type-I clathrates, especially in 6*c* sites. The experimental observed Cs_8_Sn_44_ was formed from the missing two Sn atoms in the 6*c* sites [39]. Besides, theoretical calculations by Iitaka et al. also showed that 6*c* sites lattice vacancies formation under high pressure was indeed energetically preferable. By the model of partially occupied Si sites they explained the transition pressure and change of Raman spectra of both K_8_Si_46_ and Ba_8_Si_46_ [20]. Moreover, our recent work showed that 6*c* sites lattice vacancies increased the compressibility of clathrate greatly while guest atoms vacancies hardly had any influence on this property [19]. Experimentally, by performing high quality in situ high-pressure angle-dispersive X-ray powder diffraction measurements, Li et al. found a highly disordered Si framework from analysis of the obtained anomalously large Si thermal parameters [13]. Also, present lattice dynamics calculation for K_8_Si_46_ shows that the Si-Si bond “softens” under high pressure which again provides theoretical possibility for the formation of lattice vacancies. If one considers this mechanism, the volume collapse pressure of K_8_Si_46_ is believed to be reduced. Thus, in view of these results, guest K atoms displacement induced phonon instability from earlier ab initio phonon band structure calculations [8] is indeed more likely to be caused by the disadvantages of the finite displacement method in treating these large cell calthrate compound, especially under high pressure. Our calculated results based on DFTP method obviously gives a more convincing and clear physical picture for the instability of K_8_Si_46_ under high pressure. 

### 3.2. Thermal Properties from Quasi-Harmonic Approximation

The results of calculated phonon spectra and phonon density of state can be used to compute the thermodynamic properties using the quasi-harmonic approximation (QHA) [40]. In the QHA, the phonon Helmholtz free energy is given by:

(1) $${F^{*}}_{vib}(T)=k_{B}T\int_{0}^{\infty }\left[\frac{1}{2}\hbar \omega +k_{B}T\ln(1-e^{-\frac{\hbar \omega }{k_{B}T}})\right]f(\omega )d\omega$$

where *k*_B_ is the Boltzmann constant. *ħ* is Planck’s constant and *f*(*ω*) is the phonon density of states (PDOS). Through a series calculation of PDOS of K_8_Si_46_ with different volumes, the volume dependence of Helmholtz free energy *F*_vib_(*V*,*T*) can be obtained. Then the vibrational contribution to the entropy, the specific heat at constant volume and isothermal bulk modulus can be derived by:

(2) $$S_{vib}(T)={\left(\frac{\partial F_{vib}}{\partial T}\right)}_{V}$$

(3) $$C_{v}(T)=-T{\left(\frac{\partial^{2}F_{vib}}{\partial T^{2}}\right)}_{V}$$

(4) $$B_{T}=-V(\frac{\partial P}{\partial V})T=V{\left(\frac{\partial^{2}F_{vib}}{\partial V^{2}}\right)}_{T}$$

The Grüneisen parameters can be computed by the volume derivative of (−*TS*):

(5) $$\gamma =-\frac{V}{C_{V}T}{\left(\frac{\partial (-TS)}{\partial V}\right)}_{T}$$

Then, the volume coefficient of thermal expansion and constant pressure heat capacity (*C*_p_) follows:

(6) $$\alpha_{V}=-\frac{1}{V}{\left(\frac{\partial V}{\partial T}\right)}_{P}=\frac{\gamma C_{V}}{VB_{T}}$$

(7) $$C_{P}=C_{V}(1+\gamma \alpha_{V}T)$$

**Figure 4:**
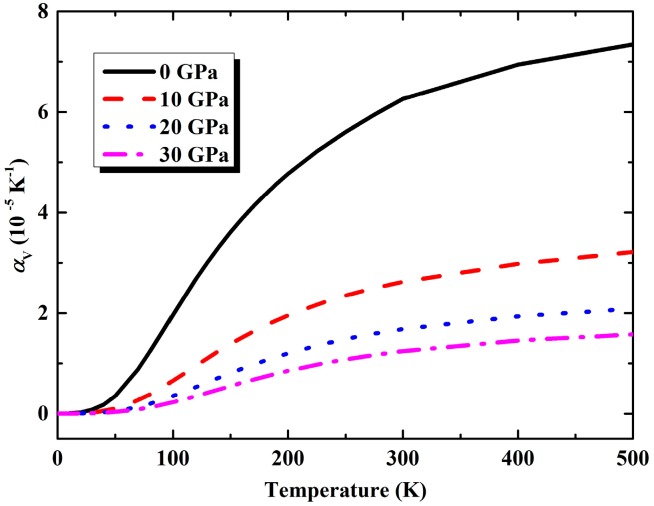
The calculated variation of volume coefficient of thermal expansion of K_8_Si_46_ with temperature under 0, 10, 20 and 30 GPa.

**Figure 5:**
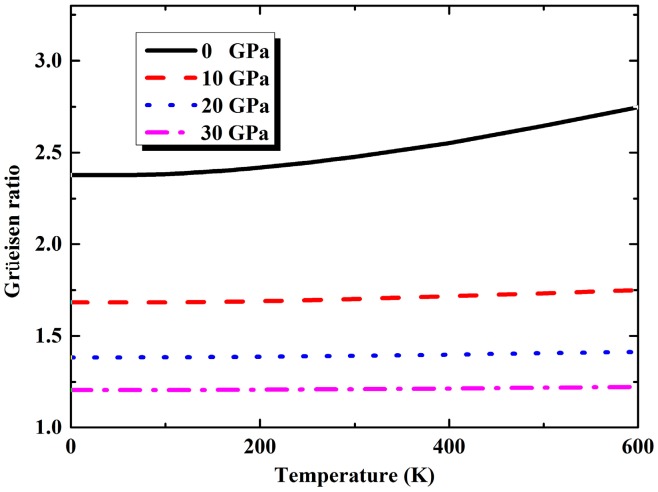
The calculated variation of Grüneisen parameters of K_8_Si_46_ with temperature under 0, 10, 20 and 30 GPa.

From QHA calculation, zero-point energy *F*_vib_ (*T* = 0) of K_8_Si_46_ compound is determined to be 2.688 eV. Moreover, the calculated variation of volume thermal expansion coefficient *α_V_* with temperature under different pressures are illustrated in Figure 4 from which we can find that *α_V_* increases rapidly with temperature below about 200 K and pressure imposes a strong restraint on the lattice expansion. At room temperature and zero pressure, the *α_V_* is predicted as 6.26 × 10^−5^ K^−1^, corresponding to a linear thermal expansion coefficient *α_L_* as 2.09 × 10^−5^ K^−1^ which is very close to the experimental value of Na_8_Si_46_ (about 2.0 × 10^−5^ K^−1^) given by Qiu et al. [41]. In addition, another important thermodynamic quantity of Grüneisen parameters which is difficult to determine experimentally can also be predicted by QHA method. The obtained temperature dependencies of Grüneisen parameters under pressure of 0, 10, 20 and 30 GPa are presented in Figure 5. It can be found that the Grüneisen parameters become almost constant in relation to the varied temperature under high pressure. At ambient conditions, the Grüneisen parameter is found to be 2.47. For congener compound Na_8_Si_46_ under same condition, this value was reported as 2.68 [41]. 

These similarities in thermdynamic properties of type I clathrates doped with same group elements have also been reported by many experimental works, for example, the thermal expansion coefficients of Ba(Sr)_8_Ga_16_Ge_30_ and Sr_8_Ga_16 _Ge_30_ [42] were just found to be almost identical to each other, and so were Rb_8_Sn_44_□_2_ and Cs_8_Sn_44_□_2_ (□ means lattice vacancy) [43]. Consequently, in general, the thermal expansion coefficient of intermetallic clathrates is assumed to mainly depend on the bonding of the framework atoms. The nature of the guest atoms just make a small contribution due to their weaker ionic bonding to the host structure. However, in the case of Ba_8_Si_46_, which has been attracting extensive attention due to the discovery of its superconductivity, the measured *α_L_* at room temperature was found to be only 1.2 × 10^−5^ K^−1^. Besides, a drop of *α_L_* of Ba_8_Si_46_ occurred at the superconducting transition temperature and the frequency-dependent Grüneisen parameter of Ba_8_Si_46_ indicated strong anharmonicity of the lattice vibrations for low energy mode with a value of up to 8.6 while higher energy modes are much less anharmonic with a value somewhat below 2 [44]. These novel results of Ba_8_Si_46_ are quite different from those of Na(K)_8_Si_46_ which reveals the non-negligible role of different hybridization between the guest and host atoms in studying the vibrational properties for doped clathrate compound. Moreover, the vacancies which are very likely to be formed in clathrate compounds can also significantly influence the system vibrational properties. Their presence can decrease the average bonding strength among host atoms and induce the displacement of guest atoms from their ideal positions. Thus, the anharmonicity of system is enhanced which would yeild an increment of the thermal expansion coefficient and Grüneisen parameters at zero pressure that had been identified by the experimental work for type-I Ge-based clathrates [45]. However, this effect became more unintelligible under high pressure because the formation of vacancies can also increase the compressbility of the clathrate which would lead the volume collapse under compress. So, further theoretical simulation with considering the formation of vacancies in hosts framework is expected to explore the effects of these vacancies on the vibrational properties of clathrate system under high pressure. 

**Figure 6:**
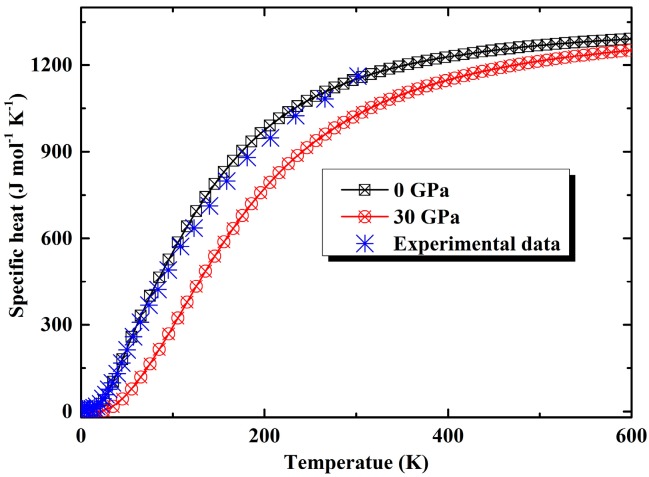
The value of *C*_P_ of K_8_Si_46_ as a function of temperature at 0 and 30 GPa, in comparison with the experimental data [41].

Because the experimental specific heat was conducted at constant pressure namely *C*_p_, in Figure 6, the resulting dependence of *C*_p_ on temperature calculated from QHA method at zero pressure is illustrated, the experimental data up to room temperature from Stefanoski have also been plotted together for a comparison [46]. It can be found that they agree with each other excellently (for instance, the specific heat at room temperature determined by present work is 1151.5 J·mol^−1^·K^−1^, experimental measured value is 1159.6 J·mol^−1^·K^−1^) which indicates the validity of present lattice dynamic simulation within DFTP. At low temperatures, the temperature dependence of specific heat presents a similar behavior to thermal expansion coefficient. When the temperature is higher than about 400 K, specific heat gradually approaches the Dulong-Petit limit, i.e., 3*nN*_A_*k*_B_ (about 1397.2 J·mol^−1^·K^−1^) which is followed by all solid at high temperatures. In our former study [47], we found the specific heat of Na_8_Si_46_ predicted by quasi-harmonic Debye model was underestimated obviously at low temperature which revealed the limitation of the Debye model in dealing with these doped clathrate compounds. The experimental heat capacities of Na_8_Si_46_ were finally reproduced by treating the special “rattle” modes of captured Na atoms in cages as Einstein oscillators. In the present work, a full phonon calculation within DFPT method can give a rather exact description of vibrational properties of these clathrate compounds which avoids a discriminatory treatment of the host lattice and encapsulated atoms. The specific heat of K_8_Si_46_ under pressure of 30 GPa is also presented in Figure 6, from which it can be found that pressure can decrease the specific heat considerably due to the suppression of lattice vibration. 

The comparison of the calculated specific heat to that predicted by the Debye model leads to the concept of the temperature dependent Debye temperature. The obtained Debye temperature at the high temperature limit is 550 K, which is consistent with the reported experimental result of 577 K [48]. In addition, the thermal conductivity *ĸ*_L_ contributed by the lattice can be estimated by the Debye equation [49]:

(8) $$\kappa_{L}=\lambda_{V}^{m}C/3$$

where *C* is the volumetric heat capacity, *λ* is the mean free path of phonons, assumed as the average distance between the guest atoms, *V*_m_ is the velocity of sound which can be derived from Debye temperature [50]. In this way, the lattice thermal conductivity is given as 1.64 W m^−1^·K^−1^ which is comparable to other reported room temperature thermal conductivities of type-I silicon based clathrate compound [48].

## 4. Conclusions

In summary, we have given a detailed study of the vibrational and thermal properties of type-I calthrate K_8_Si_46_. By employing the linear-response method within the density functional perturbation theory (DFPT), we obtained the phonon dispersion relation in K_8_Si_46_ crystal as well as phonon density of states which contained detail information about the vibrational properties of both guest atoms and framework lattice. Under a pressure of 40 GPa, the frequencies from the motions of framework silicon atoms become imaginary around the M symmetry point suggests the instability of K_8_Si_46_ crystal. The determined phonon instable pressure by lattice dynamic simulation is larger than the transition pressure observed experimentally. However, actual phase transition may be driven by multi-mechanisms which are expected to reduce the phonon instable pressure. By using quasi-harmonic approximation, the thermal properties of K_8_Si_46_ including heat specific, thermal expansion coefficient and Grüneisen parameters are predicted. From a comparison of Na_8_Si_46_, we find that the thermal properties of these type-I calthrates are very insensitive to the different nature of guest atoms in the same group of the periodic table.
